# Community case study of patient and clinician early engagement in research on multiple chronic conditions using an implementation guide

**DOI:** 10.3389/fmed.2025.1642655

**Published:** 2025-10-10

**Authors:** Constance van Eeghen, Jennifer O’Rourke Lavoie, Kairn Stetler Kelley, Georgia Brown, Beverly Canin, Juvena Hitt, Kennedy Paron, Tonica Robinson, Hanna Schweitzer, Haley Van Serke

**Affiliations:** ^1^Department of Medicine, Larner College of Medicine, University of Vermont, Burlington, VT, United States; ^2^Cancer and Aging Research Group, Duarte, CA, United States

**Keywords:** community engagement, multiple chronic conditions, research agenda, partnering implementation guide, case study

## Abstract

This case study describes the CIRCLE (Collaboratively Inspired Research Community for Learning and Engagement) project, which aimed to advance meaningful community engagement in research about multiple chronic conditions (MCCs) by co-creating a research agenda with patients, caregivers, clinicians, and researchers. The project was conducted entirely virtually, utilizing a structured process with engagement tools adapted from a prior Patient Partner Guide. It included three stages: (1) develop the virtual environment and adapt the engagement process and tools, (2) recruit and train co-creators and facilitators to test the process and tools collaboratively and make iterative improvements, and (3) implement these tools in ten-week group sessions. Project participants in the group sessions - adults with or caring for individuals with MCCs - were recruited from a previous national trial. They engaged in interactive activities such as needs assessments related to engagement, Affinity Diagrams, and Shared Lived Experiences. Results demonstrated success in two domains: (1) successful engagement in early stages of collaborative research as measured by recruitment (59 initial participants) and high levels of retention (89%) and participation (100% completion of initial assessment; 78–82% of other assessments and activities; positive qualitative evaluations) and (2) successful completion of a Partnering Guide for Research (PGR) and a prioritized research agenda for MCCs. The project is a demonstration of intentional, structured virtual engagement processes and supportive environments fostering equitable partnerships and trust while producing intended products. The PGR is a replicable implementation guide to be used by other teams seeking to integrate community voices into health research starting with a research agenda.

## Introduction

Meaningful engagement of people who are directly affected by related health conditions in the aim, design, conduct, and implementation of research is critical for effective implementation of evidence-based healthcare changes (1–3). To be meaningful, community engagement is described as the co-creation of research in which non-academics and other interest-holders (also known as stakeholders) help guide all stages of the research (4, 5) using processes, strategies, and approaches that result in agreed-upon outcomes (6). Such engagement has been shown to increase participant enrollment (7, 8), foster trust by and support of targeted communities (9, 10), generate relevant research topics (7, 9), and build connections to people or organizations outside the study team (11). Despite these documented benefits, engaging patients in early co-creation of research (developing a research agenda) remains uncommon (12).

One of the most commonly reported barriers to community engagement in healthcare research and implementation activities is the lack of structured processes to ensure equitable communication among all project participants (13). Other recurring challenges include recruiting representative patients as partners; inadequate resources to carry out patient engagement activities; difficulty retaining patients as partners over time; risk of tokenism; patients not in suitable roles; lack of feedback given to patients about results of their engagement; and patients being engaged too late (6, 8, 13–15). As engagement efforts expand, we also begin to see evidence that patient engagement done poorly can be “ineffective, demoralizing and harmful” (15).

Some of the authors (CvE, GB, JH, JL) navigated benefits and challenges of research co-creation while conducting the Patient-Centered Outcomes Research Institute (PCORI) funded Integrating Behavioral Health and Primary Care (IBH-PC) trial. The national multi-year IBH-PC trial tested a toolkit for implementing practice change to improve care for patients managing multiple chronic conditions (MCCs) (16). Those who manage MCCs are part of a large and growing population (17) for whom engagement is an important part of their care (18). Detailed methods and results of the IBH-PC trial are published elsewhere (16).

The IBH-PC trial included an engagement plan which partnered MCC patients with the research team, inspired by the requirements of the PCORI funding mechanism (19) and the principles of Lean Management (20) on which the intervention was based. Lean Management emphasizes customer (here: patient and caregiver) needs, employee (healthcare worker) involvement and continuous improvement of workflow, a method supported by numerous evaluation studies (20–22). Three patient partners (led by JL) developed one part of the trial’s intervention: a Patient Partner Guide, which contained a structured process and implementation tools to engage the trial patients in redesigning the MCC care provided by their clinics (23). Engagement with MCC partners requires awareness of specific needs: greater risk for social isolation, access barriers, mobility challenges, or stigma related to their conditions (24). Power-sharing and value given to their lived experiences support those managing MCCs in making their contributions heard (25). Engaging such partners in redesign of care or in research requires flexibility and tailored methods (26) to integrate their needs and preferences into the outcome (27).

Despite the high level of MCC community engagement on co-creation of the IBH-PC trial, project members noted important opportunities for improvement. For example, most of the research plan was already in place before patients were engaged, so the MCC community had limited input on the research questions under study. However, the IBH-PC trial also provided valuable insight into the complexities and rewards of sustained co-creation in healthcare research and implementation (23).

Building on lessons learned about engagement during the IBH-PC trial and a desire to allow people from the MCC community to have a voice in developing future research questions, the authors embarked on the CIRCLE (Collaboratively Inspired Research Community for Learning and Engagement) project. CIRCLE expanded on earlier community engagement using the structured process and tools originally developed in the IBH-PC trial. The revised process and tools were then used to engage members of the MCC community in establishing a research agenda. The purpose of this community case study is two fold: to describe the engagement processes used to conduct the CIRCLE project and to share The Partnering Guide for Research and the MCC Research Agenda. This case study approach (28) is intended to generate an in-depth understanding of community engagement using a structured process in the real-life context of establishing a research agenda. This report is a demonstration of equitable and meaningful community engagement and provides a structured process and tools that can be applied to future research activities.

## Materials, methods, context

### Project setting, participants, and structure

The CIRCLE project, a community case study, was conducted in 2020–21 entirely on remote technological platforms for conferencing (Zoom), shared project management (Basecamp), two shared documentation and communication systems (Slack and Google), and email.

CIRCLE included three stages to (1) develop the virtual environment and adapt the PPG’s structured process and tools to support engagement, (2) recruit and train facilitators; test the process and tools and make iterative improvements, and (3) implement the process and tools to engage CIRCLE members in dialogs and the co-creation of a research agenda.

CIRCLE project membership expanded with each stage. Stage 1 was led by former IBH-PC partners with the addition of patient partners from the PCORI research community. Stage 2 expanded to include more patients managing MCCs, family care partners, clinicians, and doctoral students familiar with the MCC population. Stage 3 included patients, clinic staff, and clinicians who responded to IBH-PC surveys and volunteered to be contacted after the IBH-PC trial. IBH-PC trial patient participants were adults diagnosed with two or more of the following chronic conditions: arthritis, obstructive lung disease, diabetes, heart disease (heart failure or hypertension), mood disorder (anxiety or depression), chronic pain (headache, migraine, neuralgia, fibromyalgia, or chronic musculoskeletal pain), insomnia, irritable bowel syndrome, substance use, and behavioral health needs. Any survey respondent not able to access the Internet or respond to the invitational email was necessarily excluded.

This community case study was reviewed by the University of Vermont Committees on Human Subjects and determined to be not research.

### Stage 1: develop the virtual environment and adapt the structured process and tools of engagement

Building on the IBH-PC Patient Partner Guide (23) (PPG), the project originators and other community partners serving on the IBH-PC trial identified methods to support partner inclusion, team preparation, and team orientation toward the project goal of developing a research agenda. The PPG is a printable online workbook for on-boarding and engaging patient partners managing MCCs in primary care practices’ in-person quality improvement activities. The CIRCLE Stage 1 (Planning) team identified the components of the PPG that could be applied to research activities and removed all other content (e.g., templates for contracts, spreadsheets to document progress, etc.). The remaining engagement resources formed the basis for the Partnering Guide for Research (PGR). The team then adapted the resources to online use, including a virtual platform for synchronous and asynchronous engagement within our MCC community. We sought to create an environment that supported collaboration among all members, both anticipating and responding to participant needs around equitable access, ease of use, confidentiality, and emotional safety.

**Figure 1:**
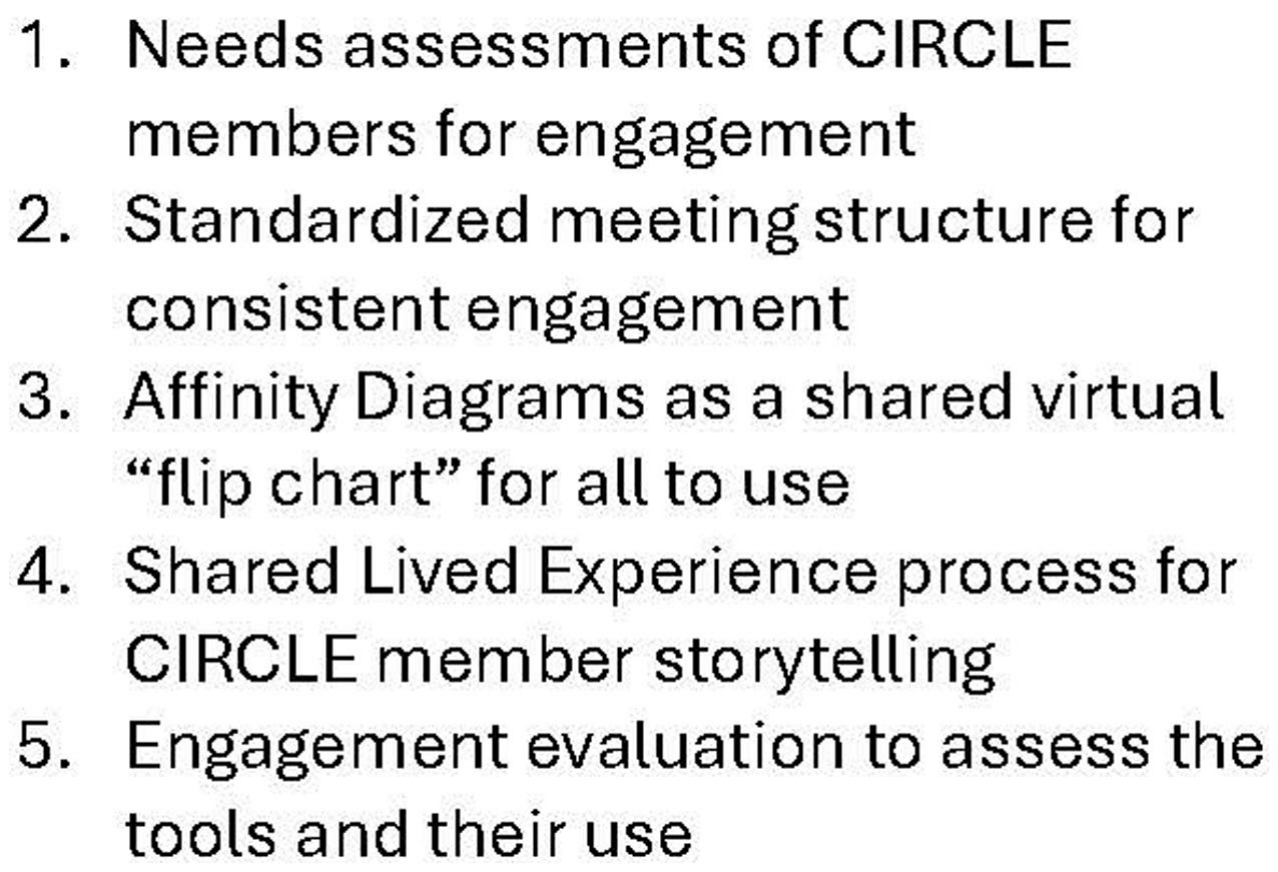
Five engagement tools. Engagement tools selected from the IBH-PC trial based on patient partner values.

We evaluated our engagement resources for alignment with IBH-PC patient partner values (23). To address common barriers to MCC engagement, we selected resources that were person-centered (29), trauma informed, easy to use, non-stigmatizing, and open to diverse lived experiences and social needs. Our team included professional and lived experience-informed experts in trauma, behavioral health, and adult education, including university-level educators, behavioral health clinicians, and researchers who engaged in monthly review sessions with the team and offered their advice to project members. With these diverse sources of guidance, we selected and refined five core tools. (Figure 1).

### Needs assessments

We developed two needs assessments to support equitable and person-centered engagement. The initial assessment (Appendix 1) gathered information on participants’ access to the virtual platforms, comfort with technology, interest in research topics, and time availability for CIRCLE group meetings. A follow up assessment (Appendix 2), completed after two meetings, sought feedback on participants’ ability to use the technology and participate in team-based activities, and identified additional support needs.

The assessments served both practical and relational purposes. They informed engagement activities and reflected the team’s commitment to person-centeredness and respect for diverse experiences. Asking about accommodations signaled a desire to know about participants’ needs, while providing support (e.g., real-time transcription) demonstrated commitment to equitable participation.

### Meeting structure

To reinforce a safe and supportive environment, CIRCLE groups followed the same structured format each week using standardized slide decks and scripts. Meetings began with a warm-up prompt with an image or quote to encourage personal reflection and sharing, followed by: (1) review of the agenda, (2) permission to record, (3) technical checks, (4) attendance, (5) review of meeting goals, (6) interactive activities to develop a MCC research agenda, and (7) preparations for the next meeting.

### Affinity diagrams

Affinity Diagrams (30) are interactive engagement tools facilitating collective answers to broad questions. Members contributed ideas, assembled ideas into themes, and captioned themes to inform a research agenda. We adapted the Google Jamboard™ application (available until December 31, 2024) to allow our groups of up to 10 participants to engage in separate meetings simultaneously using a virtual flip chart to post answers to three questions:

1. What Matters Most When You Are a Member of a Team?
2. What Matters Most When Managing Multiple Chronic Conditions?
3. What is of highest importance to you for future research that could support patients and their caregivers managing MCCs?

Members contributed first silently (writing virtual notes) and later orally (suggesting themes). This ensured all team members were able to contribute equitably by supporting different members’ participation styles according to their strengths, needs, or preferences while moving toward a research agenda.

### Shared lived experience

The Shared Lived Experience activity invited individual CIRCLE members to share a personal healthcare story related to MCCs with their group. For each CIRCLE group, one facilitator used a list of questions (Appendix 3) and a script to support storytelling, clarify key details, and guide a group discussion. A second facilitator acted as note-taker and documented: (1) the story itself, (2) the group’s discussion, and (3) lessons for research. Each shared lived experience concluded with the storyteller offering their final summary of what they wanted clinicians and researchers to learn from their story. This activity provided multiple opportunities for members to connect with others over shared values, needs and preferences while highlighting differences and unique experiences.

Each Shared Lived Experience was documented in a set of templates, reviewed by the teller for accuracy, and updated as requested. After final review, each storyteller was asked for permission to share their de-identified document on the PGR internet website and on the funding organization’s website. The tools and templates were designed to support the storytellers and create group trust as well as reveal insights about the needs of the MCC community to CIRCLE members and future learners.

### Engagement evaluation

We adapted the Critical Response (31) framework to survey collaborators during Stage 2 and CIRCLE members at the end of Stage 3 about the engagement process. Using an appreciative approach (32) with open-ended questions, the evaluation was designed to reflect project values of equity, inclusion, and transparency while providing feedback for improvement of the structured engagement process and tools. CIRCLE members shared their thoughts about what worked well and what questions, concerns, and suggestions they had for future engagement in research. The evaluation also asked if the respondent wanted to be contacted about future collaboration and engagement in research. (Appendix 4).

### Stage 2: recruit and train facilitators; test and improve the process and tools

We recruited 10 facilitators for Stage 2, including patients managing MCCs, family care partners, and doctoral students familiar with the MCC population, through a network of IBH-PC co-investigators and consultants.

After orienting facilitators to the project, facilitators began training by using the five engagement tools in the PGR. As facilitators became familiar with these tools, they refined both the tools and the scripts to guide their use. During this stage, the scripts grew to include prompts to support participation, establish team norms, manage team dynamics, respond to the emotional demands of storytelling (both on the storyteller and the listeners), facilitate confidentiality, and end on time. The scripts also provided prompts to recognize why a particular theme or story resonated and to encourage reflections from different participants on the personal relevance of these themes or stories for research or future healthcare transformation. As each tool was practiced and refined, we revised the structured process of the PGR to reflect the changes.

### Stage 3: engage MCC community members in CIRCLE project

An invitational email was sent to former IBH-PC trial participants who had consented to be contacted.

One-hour virtual information meetings were scheduled based on the availability of respondents. CIRCLE project members were offered the option to receive $25 for each 60–90 min meeting attended. Fifty-nine people attended the information meetings and completed the initial needs assessment survey; all were included as CIRCLE members (Appendix 1).

CIRCLE members (approximately 10 per group) joined one of five weekday meeting groups and remained with that group for the full 10-week project. Each group session was co-facilitated by two facilitators from Stage 2. One facilitator focused on the structure and content of the meeting, while the other supported note-taking, responded to technology needs, and attended to group dynamics. Facilitators were trained in all tasks and were free to organize their facilitation tasks in whatever way worked best for their dyad. If either facilitator was not available for a session, because of illness for example, then a trained facilitator from another group took their place.

Meetings followed the consistent structure developed in stages 1 and 2, with specific activities planned for each week. A follow up needs assessment was sent after the second meeting. (Appendix 2) The final engagement survey was sent after all Shared Lived Experiences were completed, between the 7^th^ and 9^th^ meetings. (Appendix 4).

**Figure 2:**
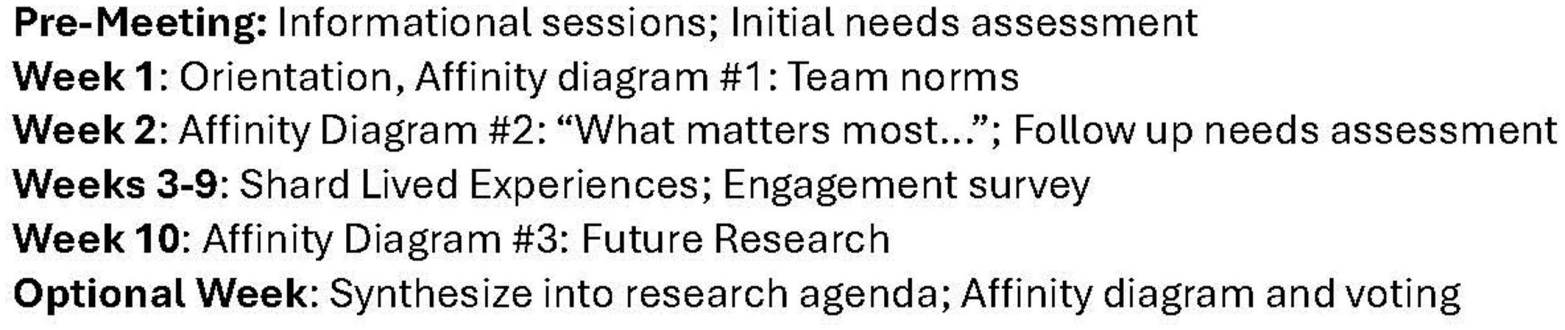
Stage 3 timeline. Activities of engagement during the 10-week community case study.

In the 10th and final meeting of each CIRCLE group, facilitators led the planned Affinity Diagram exercise to develop a research agenda for MCCs using the prompt “Based on your lived experiences and what you learned during CIRCLE, what is of highest importance to you for future research that could support patients and their caregivers managing multiple chronic health conditions?” After creating themes out of their responses, each group used “Liberating Structures” (33, 34) group problem-solving tools to determine the relationship of each theme with the others and their priority: an Interrelationship Diagraph (35) followed by Virtual Nominal Group voting (36). The resulting prioritized themes were pooled for a final group of CIRCLE members who volunteered to organize the groups’ work into the final Research Agenda in two additional meetings. All CIRCLE members were invited to join this final group, resulting in the Stage 3 Timeline (Figure 2).

### Measures and analysis

Engagement was measured by recruitment, retention, and responsiveness to surveys. We reported descriptive statistics of the two needs assessment surveys. We used Glaser’s Constant Comparative method (37) to examine the evaluation survey’s qualitative feedback, grouping similar feedback into themes, comparing new data continuously with existing data, and summarizing the results. We counted the number of respondents permitting future contact.

Products produced by this project included completion of shared lived experiences with each CIRCLE meeting group, completion of the Research Agenda for MCCs, and completion of the PGR.

## Results

### Recruitment and retention

From 1,356 potential CIRCLE partners in the IBH-PC trial, 269 individuals responded to an invitational email, 145 individuals requested meeting invitations, and 90 attended one of the informational meetings. Fifty-seven patients and 2 clinicians completed the initial needs assessment and were invited to join CIRCLE (66% of those attending the information meeting; 4% of those originally contacted).

Of the 59 patients and clinicians who joined a CIRCLE group, 51 (89%) completed the 10-week project. The eight individuals who disengaged cited the following reasons for leaving: time commitment greater than expected (4); uninterested or unwilling to engage (2); and technology challenges (1). One person did not offer a reason.

CIRCLE members represented a geographically diverse group across 13 US states and 5 time zones, with 19% from California, 13% each from Georgia, Massachusetts, and Vermont, 11% from Idaho, 7% from Washington, 6% from Kentucky, Ohio, and Oregon, and 2% each from Alaska, Hawai’i, New York, and Texas. No other demographic data were collected from CIRCLE members.

### Needs assessments

The initial needs assessment revealed strong readiness and enthusiasm among CIRCLE members for virtual engagement. All 59 members (100%) were willing to review training videos about using remote platforms, review and contribute to electronic documents, and respond to brief surveys, with 98% also open to using an online discussion board.

Accessibility needs were reported by 6 CIRCLE members, including hearing challenges (n = 2), difficulty sitting for long periods, slow Internet speed, a learning disability, and anticipated schedule changes. Overall, members reported high comfort with technology: 89% endorsed a high level of comfort with searching the Internet, 64% for typing in online documents, and 58% in learning new digital skills. Interest in project topics was also high, with 79% endorsing high interest in cultural competency, followed by healthcare and research (74%), relationship dynamics (73%), and team dynamics (54%).

In week 2, 46 of 59 (78%) completed the follow-up needs assessment. Most respondents reported being able to use video conferencing (98%), contribute to online discussion (89%), and participate in interactive team-based tools (81%). Concerns were voiced about the chat function, ability to see everyone, accessing interactive activities, and understanding how the interactive activities were related to the project goals. Regarding team dynamics, 89% indicated others “often” or “always” listened to them respectfully, 80% felt comfortable sharing with the team, and 78% felt confident contributing. Facilitators or project staff followed up with members individually as needed to address access and support team relationships.

### Meeting structure

All five CIRCLE groups completed 10 weekly meetings using the standardized meeting structure. Facilitators, assisted by their scripts, supported all CIRCLE members in accessing and navigating the online interactive tools.

### Affinity diagrams

**Figure 3:**
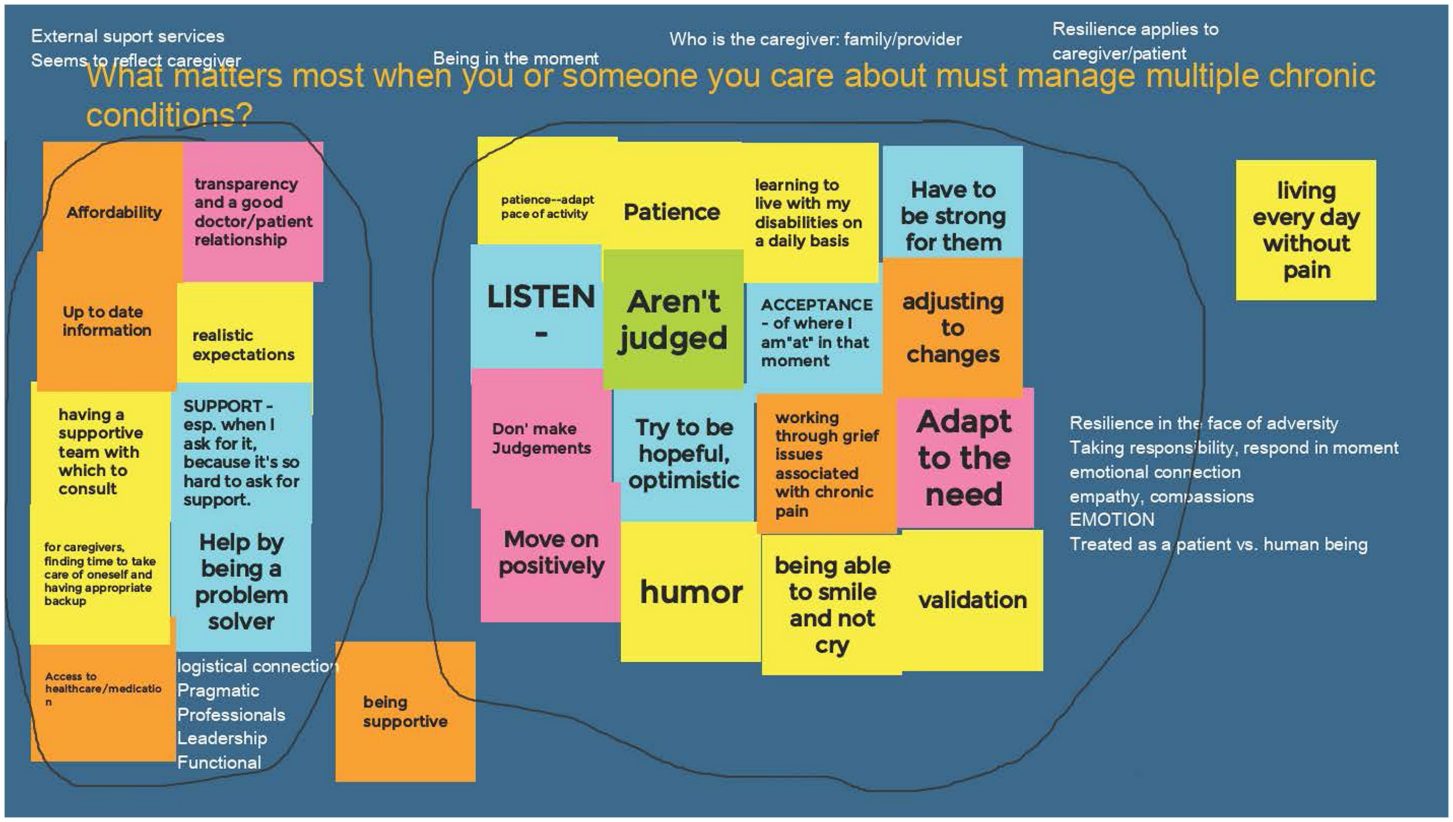
Example of a completed affinity diagram. Each group of CIRCLE members completed an affinity diagram answering the question: “What matters most when you or someone you care about must manage multiple chronic conditions?” Step 1: each member added virtual “post-it” notes to the shared web-page (colored blocks). Step 2: the members sorted the notes into groups that were related to each other. Step 3: the members discussed the groups and encircled them (black lines) to identify shared ideas. Step 4: members discussed concepts that resonated (white text added above or near groups).

Each group responded to the three Affinity Diagram questions in their meetings. When technology failed either due to signal strength or device failure, members and facilitators coordinated by zoom chat or phone to ensure that everyone’s ideas were represented. Across 5 CIRCLE Groups, 15 total diagrams were completed (Figure 3). Affinity Diagrams were posted to a website accessible to CIRCLE members for continued team work, individual reflection, and co-learning in meetings. The Affinity Diagram tool was also used in the optional week to synthesize research topics from the 5 groups into themes that could then be prioritized by Virtual Nominal Group voting (36).

### Shared lived experience

CIRCLE members shared 41 stories (80% participation, with 10 partners declining) using the Shared Lived Experience questions (Appendix 3) to tell their stories, engage in facilitated dialog with the group, and reflect on what medical professionals, researchers, and the MCC community might learn about managing multiple chronic health conditions. With 1–3 members abstaining from storytelling in each group, there was consistent but not universal engagement in this activity. Most CIRCLE members were willing to participate and found the environment safe for disclosure. When time allowed, some members shared more than one story.

### Engagement evaluation

After all groups had completed their Shared Lived Experience activities (weeks 7–9), CIRCLE members received an evaluation survey on the engagement process with open-ended requests on what worked well and what questions, concerns, and suggestions they had. Of the 51 CIRCLE members who completed the project, 42 (82%) returned their surveys and of these, 40 (95%) were willing to be contacted for further engagement beyond the 10 week period.

Comments in response to “What worked well” indicated that the process was supportive of engagement in the work of setting a research agenda by people managing multiple chronic conditions and sharing a diverse set of experiences. One participant noted “*We go up & down the scale of ages, which is a real plus. We all have had “good/not so good” events happen to us. This has helped us a great deal in understanding each other, bringing us closer,*” and another “*Our team is a vast set of persons who have a lot in common…*” and “*The participants all seem committed and cooperative, respectful of each other and eager to contribute*”.

The tools were seen as effective and supportive of the group’s work, with observations including “*The group as a whole seems to really be getting something out of the experience*” and another “*Participation is excellent and getting more open every week*,” and “*Group questions are a good design. They elicit a useful and wide-ranging discussion*”.

In response to the invitation to share concerns, individuals noted struggles with technology and maintenance of personal boundaries in group sharing activities: “*Some advice-giving responses that might not be appropriate. [The facilitator] reminded us not to do that, but for some it’s habitual*.” Other responses indicated concern about the loss of group membership at the close of the project: “*It is going to be a bit sad not to “see” these folks in person every week.*”

Although the intention was to elicit feedback about how the CIRCLE project could be improved, several respondents shared concerns focused on the challenge of influencing the health care system. For example: *“I’m scared for people like me. I’m unique in the sense that I do not fit in the same box as you or someone else. I feel like I have to fight to be heard. I should not have that level of anxiety in my life. That just adds to the hurdles we have to overcome. It’s exhausting and when does it stop? When can I feel like I can stop fighting for my rights as a patient?”* A similar comment remarked “*My only concern is if the medical staff and doctors will actually listen to what we are saying*!”

We summarized responses to “What questions do you have?” (Table 1) as opportunities to improve the engagement process. Six respondents had questions about the impact of the project on the medical community caring for people with MCCs. Remaining questions focused on next steps (5 respondents), more clarity on the use of project outcomes (5), opportunities for continued involvement (2), and questions about the engagement tools (2). These contributions were carried forward into the work of finalizing the Research Agenda for MCCs (see “Project Outcomes,” below). Additional suggestions (Appendix 5) included ideas for improvement, some of which overlapped with questions asked in Table 1. Many (33% of suggestions) provided direct feedback to specific engagement tools or processes; these were used to update the PGR.

**Table 1:** Responses to “What questions do you have about our CIRCLE project?”

Questions on…	# (%)	Summary of respondent comments
None/All good	22 (52)	
Impact on Medical Community	6 (14)	How will project be shared with healthcare professionals; how will feedback from healthcare professionals be gathered? How will findings be communicated so healthcare professionals will respond more appropriately to individuals with MCCs? Did what we do make an impact? How does our input affect medical staff and will there be another study based on this? Is our work of value to researchers? What are the useful lessons to be learned for medical professionals?
Next Steps	5 (12)	Receiving copies of the final report Presentation of the end product to funder Next, steps and examples of patient-centered research Follow-up on use of this work Follow-up on final outcome of this work How will project be shared with healthcare professionals; how will feedback from healthcare professionals be gathered?
Clarity on use of project outcomes	5 (12)	Not sure of what project is about How will stories form a conclusion? How will stories improve healthcare? How do stories answer a question? Will stories be used as a database or as part of a way to collect data?
Continued involvement	2 (5)	How to contribute more “lived experiences”? How to be involved in next study on MCCs?
Engagement tools	2 (5)	Would we be better able to gage how much a story reflected the experience of others in the group with more structure to the group’s dialog after hearing the story? Is the meeting structure too repetitious in sharing lived experiences?

### Research agenda

**Figure 4:**
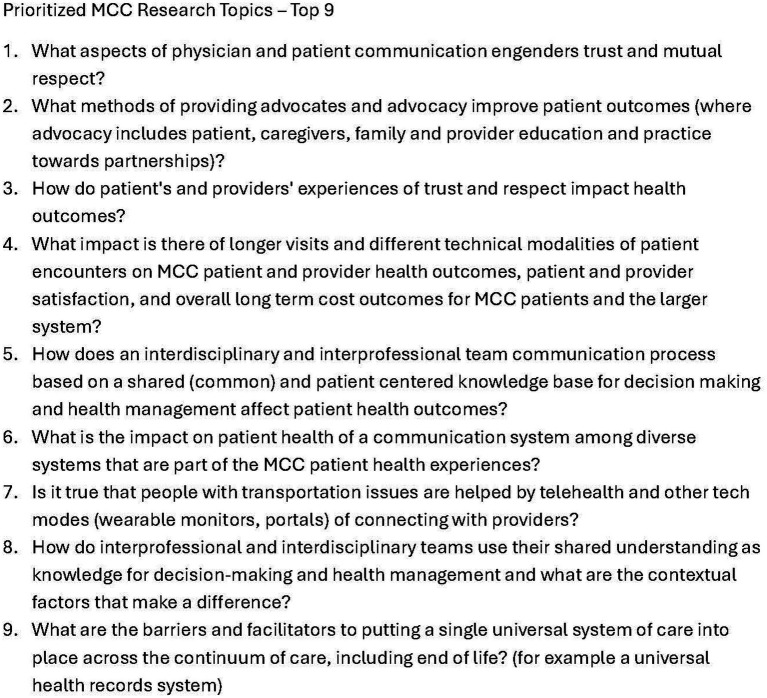
Prioritized research agenda for multiple chronic conditions. The final list of research agenda items created using the structured engagement process of the research guide for partnering.

During the 10th meeting of each CIRCLE group, members produced a total of 30 themes (minimum of 5 themes per group and maximum of 10). One to 4 CIRCLE members from each group (*n* = 14, 27%) volunteered to continue working after the 10th meeting. These volunteers joined the project team, which included researchers, patients, family members, facilitators, and clinicians, to assemble the five groups’ final Affinity Diagram themes into a single research agenda for the project. They met for two additional one-hour sessions to diagram and prioritize, using Virtual Nominal Group Voting (36), the final list of nine research topics that were consistently identified from the CIRCLE #10 meetings to create the research agenda. (Figure 4).

### Partnering guide for research

**Figure 5:**
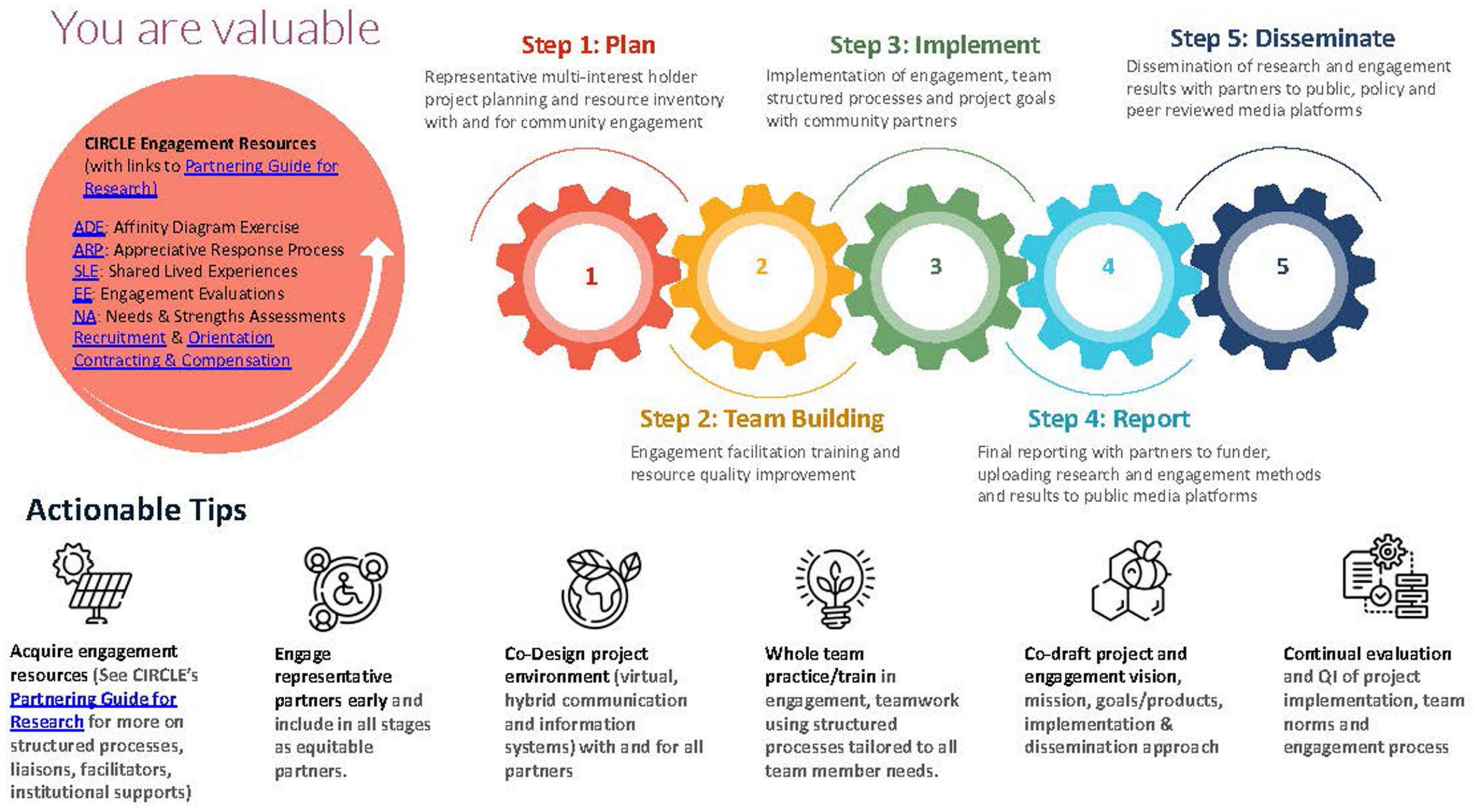
Structured process for community engagement in research. The overarching methodology of the partnering guide for research.

All final project materials, including the research agenda, were uploaded to a free website designed to help engage community members in the work of research and agenda development: The Partnering Guide for Research. The website was shared with all CIRCLE members and posted on the funding agency’s repository of engagement resources website. (December 2021) See Figure 5 for an overview of the engagement method.

## Discussion

The CIRCLE project provided a community case study of sustainable, successful engagement to produce a research agenda using a structured process and tools for building relationships and co-producing meaningful products among diverse partners. In using the methods of the PGR to improve engagement, we saw each stage of this project grow directly out of what was learned from interest-holders in earlier stages. From the starting point of a guide for the IBH-PC trial and throughout iterative revisions, the PGR reflects the contributions of over 70 community partners and can be adapted to serve the engagement needs of many other teams.

We achieved meaningful community engagement based on recruitment retention, attention to CIRCLE members’ needs, completion of engagement activities, development of a research agenda relevant to those managing MCCs, and the publication of the PGR. We demonstrated that a structured process and engagement tools can be used to engage people with MCCs across time-zones based on a commitment to being responsive to supporting participants’ needs. Examples of this commitment are found in planning for ongoing accessibility (e.g., live captioning) and helping in the moment (e.g., facilitating collaborative interactions; finding alternate ways of accessing activities if technology fails). The importance of a commitment to supporting the needs of patient partners is also identified in the Canadian Institutes of Health Research Patient Engagement Framework (38), which highlights flexible environments, along with other organizations established to promote patient and public involvement (PPI) such as INVOLVE in the U. K. and PCORI in the U. S. (39) While the value of a commitment to PPI is noted in several studies (40, 41) and posited in a recent training program based on needs assessments of research partners (42), this case study illustrates how such a commitment can be conducted.

We also achieved meaningful engagement by asking community members to work on something that matters to them through co-creation of a work product in a safe environment. The Affinity Diagram process gave all voices value; the Shared Lived Experience process provided a safe and respectful process for telling, listening to, and learning from team members’ experiences (co-learning). The repeated cycles of gathering permission, conducting an activity, listening to feedback, reflecting, and co-editing the work fostered respectful ways to collaborate in teamwork that resulted in the research agenda and the PGR. This structured process approach for inclusion of all perspectives can be found in the literature but is limited to the use of evaluation tools for patient engagement (43, 44). Although “inclusion” is featured in definitions of “patient engagement in research,” (45) the use of structured processes to accomplish such a goal is not.

CIRCLE members expressed a high level of interest at the outset of the project and responded to increasingly demanding tasks over 10 weeks. The majority of participants sustained this high level of interest through its completion. There are likely many reasons supporting their high retention rate and involvement, with some of those due to the engagement process, hearing about the lived experiences of others like them, and a desire to help others successfully meet difficult circumstances they had encountered in the past – whether as fellow patients or clinicians. With 95% willing to maintain contact with the project team in the future and the expression of sadness about the group ending (see Engagement Evaluation, above), it may be that engagement cultivates a sense of belonging, which in turn rewards engagement (46).

### Engagement priorities

We confirmed that inclusion of diverse partners, development of a supportive environment, and use of consistent methods to build capacity to do meaningful work together were the essential priorities of this engagement project. With the diversity of MCCs managed by CIRCLE members, including their varying backgrounds and locations, they were able to engage and find agreement on a research agenda that could affect many lives in important ways. This engagement process stimulated a quarter of the CIRCLE members to continue beyond their 10-week commitment, continuing to work on the research agenda, dissemination, and development of new projects, as well as meeting socially online, further strengthening those supportive relationships.

Both the technology and the meeting structure contributed to a supportive environment for engagement. By assessing technological needs and team dynamics, the project team and facilitators worked continually to remove barriers and promote the values of their work: person-centered, open to diverse lived experiences, and responsive to differing needs. Per Shippee, patient and service user initiation, reciprocal relationships, co-learning, reassessment, and feedback are integral components of engagement (14). In our project, the facilitators were at the core of sustaining the structure of group meetings and real-time problem solving to ensure the success of each CIRCLE member. Stage 2 training time was an investment that supported the success of Stage 3 and the relationships that continued beyond project end.

### Technology

Remote technology to support virtual platforms is an important component of successful engagement in our era. These platforms offer both advantages and challenges. Among its many advantages, technology provides widespread access and houses its own resources for training, if offered with support. This project took place during 2020–21, when responses to the COVID 19 pandemic included a greater dependency on and therefore acceptability of remote tools for engagement and teamwork. This likely improved participation in a project drawing together patients and other interest-holders across the U. S. In addition to wide-spread access, technology platforms can also offer equity, with appropriate facilitation, so that every participant is able to influence outcomes. Participants need to have full ability to determine the degree to which they participate (visual, audible, chats, or silent observation), which may require financial support for those without access to Internet-accessible devices. A large body of evidence supports the benefits of remote technology for engagement in patient care (47–49) and we suspect that this same relationship will hold for engagement in patient-centered research.

Perhaps the greatest technology-related challenge experienced in this engagement project was the commitment and time needed to provide prompt responsiveness and outreach of project leaders and facilitators to maintain the personal connection of team relationships. Technology can conceal, as well as reveal, the need for help. Leaders and partners alike must watch for dynamics needing attention, such as withdrawal, anger, silence, and other symptoms of disengagement or imbalances in power that can become barriers to reciprocal relationships and effective team communication.

### Sustainability of relationships

In addition to the team development needed to support research studies, it is also likely that ongoing engagement, before and after the study, is important and is part of team maintenance. The value of maintaining relationships is re-iterated in PPI guidance in the European Alliance of Associations for Rheumatology (EULAR) recommendations, the Core Outcome Measures in Effectiveness Trials (COMET) checklist, the Guidance for Reporting Involvement of Patients and the Public (GRIPP) checklist (50) and other sources (51). Research constantly builds on past discoveries and identifies new opportunities that appear on the horizon of what is known. To engage in those opportunities nimbly, interest-holder relationships are best if continually nourished. We built on responses from IBH-PC respondents as to whether they would be willing to be contacted for future projects. Establishing these relationships and finding ways to sustain them (e.g., periodic updates, evaluating future research opportunities, conducting surveys) are all part of nurturing the team and preparing it for future work that could improve health services and health.

### Creation of a research agenda for MCCs

Over a 5-month period, our partners created a research agenda with nine research agenda questions of interest to the MCC community. The Agency for Health Research and Quality (AHRQ) published a research agenda in 2021 to “guide future investments in improving MCC care.” The AHRQ research agenda identified eight domains of interest using a process that included over 150 people in stages over a 2-year period (52). Each of the nine agenda items developed by the CIRCLE project can be associated with an AHRQ domain. For example, CIRCLE agenda item #1 (communication engendering trust and mutual respect) fits into the domain of AHRQ #3 (Breaking down the clinic walls to “enable better communication with the care team and capture preferences, values, goals, and progress toward attaining them”) but adds specificity about what that means to the patient. In general, CIRCLE items are oriented around the patient’s perspective. Other CIRCLE items (e.g., #2, advocacy) were reflected broadly in AHRQ agenda but not specifically matched. Overall, the CIRCLE agenda items are at the level of a research question for a future study; the AHRQ agenda items are high level research areas and would have to be refined into a research question to be studied. An implementation strategy must match its purpose. CIRCLE’s approach matches its purpose to create and test a partnering guide for research on MCCs using structured processes to engage community members in establishing a prioritized research agenda.

### Limitations

There are several limitations to this project. We could include only those IBH-PC survey respondents who were able to respond by email and attend an informational session. This eliminated participants who did not have access to electronic communication methods or self-selected out due to real or perceived access barriers. Clinical team recruitment (2/176 [1%]) was low relative to patient recruitment (57/1180 [5%]), likely due to the intense pressure placed on primary care clinics in the then-current COVID 19 environment. There may also have been barriers we were not aware of such as alignment with personal interests or compensation as well as ethical considerations in the degree to which partners in research are treated equitably by system structures, such as payments (as salaried faculty or staff vs. temporary employees or as volunteers). It was outside the scope of this project to test whether community engagement results in more implementable research. We did learn how to make engagement in research more implementable.

### Conclusion

The CIRCLE project demonstrated that structured, virtual co-creation processes can meaningfully involve patients, caregivers, and clinicians in research agenda-setting for multiple chronic conditions (MCCs). By adapting and iteratively refining a structured engagement process and tools -- such as needs assessments, Affinity Diagrams, Shared Lived Experiences, and other collaborative activities -- the project fostered equitable participation, built trust, and enabled diverse voices to shape research priorities. Use of these engagement resources, with different teams and different facilitators at different times, demonstrated that these online structured processes can engage people who manage multiple chronic conditions. High retention and active participation across geographically dispersed groups suggest that the approach was both accessible and effective in supporting sustained collaboration. The resulting Partnering Guide for Research provides a replicable implementation method for future initiatives seeking to center community perspectives in research activities. Ultimately, CIRCLE’s experience highlights that intentional design of diverse partnerships, a supportive environment, and responsive, consistent facilitation to engage in meaningful work are critical for achieving successful community engagement in health research.

## Data Availability

The original contributions presented in the study are included in the article/Supplementary material, further inquiries can be directed to the corresponding author.

## Author’s note

The authors are a multi-interest-group, which includes patients, family caregivers, clinicians, patient advocates, and researchers.

## Author disclaimer

The views, statements, and opinions presented in this report are solely the responsibility of the author(s) and do not necessarily represent the views of the Patient-Centered Outcomes Research Institute (PCORI), its Board of Governors or Methodology Committee. The Patient-Centered Outcomes Research Institute (PCORI) is an independent, nonprofit organization authorized by Congress in 2010. Its mission is to fund research that will provide patients, their caregivers, and clinicians with the evidence-based information needed to make better-informed healthcare decisions. PCORI is committed to continually seeking input from a broad range of stakeholders to guide its work.

## References

[1] Maurer M Mangrum R Hilliard-Boone T Amolegbe A Carman KL Forsythe L . Understanding the influence and impact of stakeholder engagement in patient-centered outcomes research: a qualitative study. J Gen Intern Med. (2022) 37:6–13. doi: 10.1007/s11606-021-07104-w, PMID: 35349017 PMC8993962

[2] Dy T Hamilton WJ Kramer CB Apter A Krishnan JA Stout JW . Stakeholder engagement in eight comparative effectiveness trials in African Americans and Latinos with asthma. Res Involv Engagem. (2022) 8:63. doi: 10.1186/s40900-022-00399-x, PMID: 36434672 PMC9694541

[3] Maurer ME Lavelle ME Hilliard-Boone T Frazier K Agraviador D Mosbacher R . Researcher and partner perspectives on the relationship between engagement in research and three uses of patient-centered comparative clinical effectiveness research study findings. Res Involv Engagem. (2024) 10:105. doi: 10.1186/s40900-024-00632-9, PMID: 39402691 PMC11476073

[4] World Health Organization. *Community engagement: a health promotion guide for universal health coverage in the hands of the people*. Geneva: World Health Organization. (2020). Available online at: https://www.who.int/publications/i/item/9789240010529.

[5] Halvorsrud K Kucharska J Adlington K Rüdell K Brown Hajdukova E Nazroo J . Identifying evidence of effectiveness in the co-creation of research: a systematic review and meta-analysis of the international healthcare literature. J Public Health. (2019) 43:197–208. doi: 10.1093/pubmed/fdz126, PMID: 31608396 PMC8042368

[6] Aguilar-Gaxiola S Ahmed SM Anise A Azzahir A Baker KE Cupito A . Assessing meaningful community engagement: a conceptual model to advance health equity through transformed systems for health: organizing committee for assessing meaningful community engagement in health and health care programs and policies. NAM Perspect. (2022) 2022:202. doi: 10.31478/202202c PMC9303007 35891775

[7] Vat LE Finlay T Jan Schuitmaker-Warnaar T Fahy N Robinson P Boudes M . Evaluating the "return on patient engagement initiatives" in medicines research and development: a literature review. Health Expect. (2020) 23:5–18. doi: 10.1111/hex.12951, PMID: 31489988 PMC6978865

[8] Domecq JP Prutsky G Elraiyah T Wang Z Nabhan M Shippee N . Patient engagement in research: a systematic review. BMC Health Serv Res. (2014) 14:89. doi: 10.1186/1472-6963-14-89, PMID: 24568690 PMC3938901

[9] Sheridan S Schrandt S Forsythe L Hilliard TS Paez KA. The PCORI engagement rubric: promising practices for partnering in research. Ann Fam Med. (2017) 15:165–70. doi: 10.1370/afm.2042, PMID: 28289118 PMC5348236

[10] Hovén E Eriksson L Månsson D'Souza Å Sörensen J Hill D Viklund C . What makes it work? Exploring experiences of patient research partners and researchers involved in a long-term co-creative research collaboration. Res Involv Engagem. (2020) 6:33. doi: 10.1186/s40900-020-00207-4, PMID: 32579132 PMC7305606

[11] Lansing AE Romero NJ Siantz E Silva V Center K Casteel D . Building trust: leadership reflections on community empowerment and engagement in a large urban initiative. BMC Public Health. (2023) 23:1252. doi: 10.1186/s12889-023-15860-z, PMID: 37380973 PMC10304359

[12] Bednarek AT Miyamoto B Corbett K Hudson C Scarrow G Brass M . How and why funders support engaged research. Proc Natl Acad Sci. (2025) 122:e2400931121. doi: 10.1073/pnas.2400931121, PMID: 39793032 PMC11725928

[13] Woodward EN Castillo AIM True G Willging C Kirchner JE. Challenges and promising solutions to engaging patients in healthcare implementation in the United States: an environmental scan. BMC Health Serv Res. (2024) 24:29. doi: 10.1186/s12913-023-10315-y, PMID: 38178131 PMC10768202

[14] Shippee ND Domecq Garces JP Prutsky Lopez GJ Wang Z Elraiyah TA Nabhan M . Patient and service user engagement in research: a systematic review and synthesized framework. Health Expect. (2015) 18:1151–66. doi: 10.1111/hex.12090, PMID: 23731468 PMC5060820

[15] Richards DP Poirier S Mohabir V Proulx L Robins S Smith J. Reflections on patient engagement by patient partners: how it can go wrong. Res Involv Engagem. (2023) 9:41. doi: 10.1186/s40900-023-00454-1, PMID: 37308922 PMC10262483

[16] Littenberg B Clifton J Crocker AM Baldwin L-M Bonnell LN Breshears RE . A cluster randomized trial of primary care practice redesign to integrate behavioral health for those who need it Most: patients with multiple chronic conditions. Ann Fam Med. (2023) 21:483–95. doi: 10.1370/afm.3027, PMID: 38012036 PMC10681692

[17] Diederichs C Berger K Bartels DB. The measurement of multiple chronic diseases—a systematic review on existing multimorbidity indices. J Gerontol A Biol Sci Med Sci. (2010) 66A:301–11. doi: 10.1093/gerona/glq208, PMID: 21112963

[18] Vick JB Wolff JL. A scoping review of person and family engagement in the context of multiple chronic conditions. Health Serv Res. (2021) 56:990–1005. doi: 10.1111/1475-6773.13857, PMID: 34363217 PMC8515220

[19] PCORI Engagement Rubric. *PCORI (Patient-Centered Outcomes Research Institute)*. (2014). Available online at: https://www.pcori.org/document/engagement-rubric.

[20] Lawal AK Rotter T Kinsman L Sari N Harrison L Jeffery C . Lean management in health care: definition, concepts, methodology and effects reported (systematic review protocol). Syst Rev. (2014) 3:103. doi: 10.1186/2046-4053-3-103, PMID: 25238974 PMC4171573

[21] Klein LL Vieira KM Feltrin TS Pissutti M Ercolani LD. The influence of lean management practices on process effectiveness: a quantitative study in a public institution. SAGE Open. (2022) 12:88837. doi: 10.1177/21582440221088837

[22] Sinha N Matharu M. A comprehensive insight into lean management: literature review. J Ind Eng Manag. (2019) 12:302–17. doi: 10.3926/jiem.2885

[23] van Eeghen C Hitt JR Pomeroy DJ Reynolds P Rose GL O'Rourke Lavoie J. Co-creating the patient partner guide by a multiple chronic conditions team of patients, clinicians, and researchers: observational report. J Gen Intern Med. (2022) 37:73–9. doi: 10.1007/s11606-021-07308-0, PMID: 35349025 PMC8960693

[24] Blickem C Kennedy A Vassilev I Morris R Brooks H Jariwala P . Linking people with long-term health conditions to healthy community activities: development of patient-led assessment for network support (PLANS). Health Expect. (2013) 16:e48–59. doi: 10.1111/hex.12088, PMID: 23731452 PMC3908360

[25] AuYoung M Fagan P Rhoads KF. Operationalizing meaningful community engagement to reduce the burden of multiple chronic conditions. Am J Public Health. (2025) 115:S92–3. doi: 10.2105/AJPH.2025.308179, PMID: 40561409 PMC12199680

[26] Markle-Reid M Ganann R Ploeg J Heald-Taylor G Kennedy L McAiney C . Engagement of older adults with multimorbidity as patient research partners: lessons from a patient-oriented research program. J Multimorb Comorb. (2021) 11:99508. doi: 10.1177/2633556521999508, PMID: 33796472 PMC7975523

[27] Bayliss EA Bonds DE Boyd CM Davis MM Finke B Fox MH . Understanding the context of health for persons with multiple chronic conditions: moving from what is the matter to what matters. Ann Fam Med. (2014) 12:260–9. doi: 10.1370/afm.1643, PMID: 24821898 PMC4018375

[28] Crowe S Cresswell K Robertson A Huby G Avery A Sheikh A. The case study approach. BMC Med Res Methodol. (2011) 11:100. doi: 10.1186/1471-2288-11-100, PMID: 21707982 PMC3141799

[29] Woo SE Jebb AT Tay L Parrigon S. Putting the “person” in the Center:review and synthesis of person-centered approaches and methods in organizational science. Organ Res Methods. (2018) 21:814–45. doi: 10.1177/1094428117752467

[30] Harboe G Huang EM. *Real-world affinity diagramming practices: bridging the paper-digital gap*. CHI '15: Proceedings of the 33rd Annual ACM Conference on Human Factors in Computing Systems, pp. 95–104. (2015).

[31] Lerman L Borstel J. Critical response process: A method for getting useful feedback on anything you make, from dance to dessert. Maryland: Dance Exchange, Inc. (2003).

[32] Stowell F. The appreciative inquiry method—a suitable candidate for action research? Syst Res Behav Sci. (2013) 30:15–30. doi: 10.1002/sres.2117

[33] Mahoney JS Lewin L Beeber L Willis DG. Using liberating structures to increase engagement in identifying priorities for the APNA research council. J Am Psychiatr Nurses Assoc. (2016) 22:504–7. doi: 10.1177/1078390316663308, PMID: 27519614

[34] Lipmanowicz H Singhal A McCandless K Wang H. Liberating structures: Engaging everyone to build a good life together In: Wang H, editor. Communication and “the good life”. New York: Peter Lang (2015). 233–46.

[35] AABB. *Quality toolkit |interrelationship diagram*. Bethesda, MD: Association for the Advancement of Blood and Biotherapies. (2025). Available online at: https://www.aabb.org/docs/default-source/default-document-library/resources/quality-tool-interrelationship-diagram.pdf.

[36] Lee SH Ten Cate O Gottlieb M Horsley T Shea B Fournier K . The use of virtual nominal groups in healthcare research: an extended scoping review. PLoS One. (2024) 19:e0302437. doi: 10.1371/journal.pone.0302437, PMID: 38865305 PMC11168680

[37] Glaser B. The constant comparative method of qualitative analysis. Soc Probl. (1965) 12:436–45. doi: 10.2307/798843

[38] The Canadian Institutes of Health Research. *Strategy for patient-oriented research: patient engagement research*. The Canadian Institutes of Health Research (2019). Available online at: https://cihr-irsc.gc.ca/e/documents/spor_framework-en.pdf.

[39] McCarron TL Clement F Rasiah J Moran C Moffat K Gonzalez A . Patients as partners in health research: a scoping review. Health Expect. (2021) 24:1378–90. doi: 10.1111/hex.13272, PMID: 34153165 PMC8369093

[40] Béland S Lambert M Delahunty-Pike A Howse D Schwarz C Chouinard MC . Patient and researcher experiences of patient engagement in primary care health care research: a participatory qualitative study. Health Expect. (2022) 25:2365–76. doi: 10.1111/hex.13542, PMID: 35593113 PMC9615076

[41] Lauzon-Schnittka J Audette-Chapdelaine S Boutin D Wilhelmy C Auger AM Brodeur M. The experience of patient partners in research: a qualitative systematic review and thematic synthesis. Res Involv Engagem. (2022) 8:55. doi: 10.1186/s40900-022-00388-0, PMID: 36192817 PMC9528123

[42] Terry AL Bayliss L Meredith L Law E Van Hoorn R Regan S. A training program to support patient engagement in primary health care research: co-design, implementation, and evaluation study. J Particip Med. (2025) 17:e65485. doi: 10.2196/65485, PMID: 40472265 PMC12161618

[43] Boivin A L'Espérance A Gauvin FP Dumez V Macaulay AC Lehoux P . Patient and public engagement in research and health system decision making: a systematic review of evaluation tools. Health Expect. (2018) 21:1075–84. doi: 10.1111/hex.12804, PMID: 30062858 PMC6250878

[44] Easley J Wassersug R Matthias S Tompson M Schneider ND O’Brien MA . Patient engagement in health research: perspectives from patient participants. Curr Oncol. (2023) 30:2770–80. doi: 10.3390/curroncol30030210, PMID: 36975423 PMC10047022

[45] Harrington RL Hanna ML Oehrlein EM Camp R Wheeler R Cooblall C . Defining patient engagement in research: results of a systematic review and analysis: report of the ISPOR patient-centered special interest group. Value Health. (2020) 23:677–88. doi: 10.1016/j.jval.2020.01.019, PMID: 32540224

[46] Allen KA Kern ML Rozek CS McInereney D Slavich GM. Belonging: a review of conceptual issues, an integrative framework, and directions for future research. Aust J Psychol. (2021) 73:87–102. doi: 10.1080/00049530.2021.1883409, PMID: 33958811 PMC8095671

[47] Sawesi S Rashrash M Phalakornkule K Carpenter JS Jones JF. The impact of information technology on patient engagement and health behavior change: a systematic review of the literature. JMIR Med Inform. (2016) 4:e1. doi: 10.2196/medinform.4514, PMID: 26795082 PMC4742621

[48] Alomar D Almashmoum M Eleftheriou I Whelan P Ainsworth J. The impact of patient access to electronic health records on health care engagement: systematic review. J Med Internet Res. (2024) 26:e56473. doi: 10.2196/56473, PMID: 39566058 PMC11618012

[49] Senek M Drummond D Pinnock H Hansen K Ankolekar A O'Connor Ú . Impact of digital health on patient-provider relationships in respiratory secondary care based on qualitative and quantitative evidence: systematic review. J Med Internet Res. (2025) 27:e70970. doi: 10.2196/70970, PMID: 40446293 PMC12166327

[50] Arumugam A Phillips LR Moore A Kumaran SD Sampath KK Migliorini F . Patient and public involvement in research: a review of practical resources for young investigators. BMC Rheumatol. (2023) 7:2. doi: 10.1186/s41927-023-00327-w, PMID: 36895053 PMC9996937

[51] Auwal FI Copeland C Clark EJ Naraynassamy C McClelland GR. A systematic review of models of patient engagement in the development and life cycle management of medicines. Drug Discov Today. (2023) 28:103702. doi: 10.1016/j.drudis.2023.103702, PMID: 37453460

[52] Bierman AS Wang J O'Malley PG Moss DK. Transforming care for people with multiple chronic conditions: Agency for Healthcare Research and Quality's research agenda. Health Serv Res. (2021) 56:973–9. doi: 10.1111/1475-6773.13863, PMID: 34378192 PMC8515222

