# Supplementary material for: Community case study of patient and clinician early engagement in research on multiple chronic conditions using an implementation guide
Source: Front Med (Lausanne). 2025 Oct 10;12:1642655. doi: 10.3389/fmed.2025.1642655 (PMC12549578; doi:10.3389/fmed.2025.1642655)
Supplement: Supplementary file 1 [file Data_Sheet_1.pdf]

CIRCLE Participation Survey

Please complete the survey below.

Thank you!

First Name ______________________________

Last Name ______________________________

Email address ______________________________

What Information Meeting did you attend?
- ◯ Monday January 25th 10:00-11:00 am EST
- ◯ Tuesday January 26th 8:30-9:30 pm EST
- ◯ Wednesday January 27th 4:00-5:00 pm EST
- ◯ Thursday January 28th 7:00-8:00 pm EST
- ◯ Friday January 29th 1:00-2:00 pm EST

Are you able to commit to starting CIRCLE meetings the third week of February (Monday, February 15) at 10:00-11:00 am Eastern Standard Time?
- ◯ Yes
- ◯ No

Are you able to commit to starting CIRCLE meetings the third week of February (Tuesday, February 16) at 8:30-9:30 pm Eastern Standard Time?
- ◯ Yes
- ◯ No

Are you able to commit to starting CIRCLE meetings the third week of February (Wednesday, February 17) at 4:00-5:00 pm Eastern Standard Time?
- ◯ Yes
- ◯ No

Are you able to commit to starting CIRCLE meetings the third week of February (Thursday, February 18) at 7:00-8:00 pm Eastern Standard Time?
- ◯ Yes
- ◯ No

Are you able to commit to starting CIRCLE meetings the third week of February (Friday, February 19) at 1:00-2:00 pm Eastern Standard Time?
- ◯ Yes
- ◯ No

Participation in this project includes participating in 10 weekly meetings.

Please indicate your availability for each of the 10 weeks.

Please note: All times are listed in EASTERN STANDARD TIME

Mondays - 10:00-11:00 am EST

Check all that you CAN attend:
- ☐ February 15, 2021
- ☐ February 22, 2021
- ☐ March 01, 2021
- ☐ March 08, 2021
- ☐ March 15, 2021
- ☐ March 22, 2021
- ☐ March 29, 2021
- ☐ April 05, 2021
- ☐ April 12, 2021
- ☐ April 19, 2021

Tuesdays - 8:30-9:30 pm EST<br><br>Check all that you CAN attend:	☐ February 16, 2021<br>☐ February 23, 2021<br>☐ March 02, 2021<br>☐ March 09, 2021<br>☐ March 16, 2021<br>☐ March 23, 2021<br>☐ March 30, 2021<br>☐ April 06, 2021<br>☐ April 13, 2021<br>☐ April 20, 2021
Wednesdays - 4:00-5:00 pm EST<br><br>Check all that you CAN attend:	☐ February 17, 2021<br>☐ February 24, 2021<br>☐ March 03, 2021<br>☐ March 10, 2021<br>☐ March 17, 2021<br>☐ March 24, 2021<br>☐ March 31, 2021<br>☐ April 07, 2021<br>☐ April 14, 2021<br>☐ April 21, 2021
Thursdays - 7:00-8:00 pm EST<br><br>Check all that you CAN attend:	☐ February 18, 2021<br>☐ February 25, 2021<br>☐ March 04, 2021<br>☐ March 11, 2021<br>☐ March 18, 2021<br>☐ March 25, 2021<br>☐ April 01, 2021<br>☐ April 08, 2021<br>☐ April 15, 2021<br>☐ April 22, 2021
Fridays - 1:00-2:00 pm EST<br><br>Check all that you CAN attend:	☐ February 19, 2021<br>☐ February 26, 2021<br>☐ March 05, 2021<br>☐ March 12, 2021<br>☐ March 19, 2021<br>☐ March 26, 2021<br>☐ April 02, 2021<br>☐ April 09, 2021<br>☐ April 16, 2021<br>☐ April 23, 2021
We hope that our CIRCLE partners will engage with us as much as you would like to in between meetings on Slack. However, there will be updates and requests made on Slack that we will need you to respond to promptly. Can you commit to participating in about 15 minutes of required Slack activities in between meetings?	○ Yes<br>○ No

Activities in Slack might include:

	Yes	No
Basic Slack training videos	◯	◯
Reviewing and providing your insight about a document	◯	◯
Responding to a brief survey	◯	◯
Responding to discussions on Slack	◯	◯

Accessibility needs

	Yes	No
Hearing challenges-do you need closed captioning to participate?	◯	◯
Do you have other circumstances that would challenge your ability to participate?	◯	◯

Can you explain: ______________________________

Please rate your comfort level for:

	Little comfort	Moderate comfort	High comfort
Typing in an online document	◯	◯	◯
Learning new computer and technical skills	◯	◯	◯
Searching the Internet	◯	◯	◯

Please let us know your interest level for:

	Little interest	Moderate interest	High interest
Learning about healthcare and research	◯	◯	◯
Learning about team dynamics	◯	◯	◯
Learning about "cultural competency" (the ability to understand, communicate with and effectively interact with people across cultures)	◯	◯	◯
Learning about relationship dynamics	◯	◯	◯
