# Supplementary material for: Community case study of patient and clinician early engagement in research on multiple chronic conditions using an implementation guide
Source: Front Med (Lausanne). 2025 Oct 10;12:1642655. doi: 10.3389/fmed.2025.1642655 (PMC12549578; doi:10.3389/fmed.2025.1642655)
Supplement: Supplementary file 2 [file Data_Sheet_2.pdf]

# CIRCLE Needs Assessment - February

Please complete the survey below.

Thank you!

---

Introduction:
One of our goals is to help future research teams engage stakeholders in research projects based on what we learn together. Our activities in Zoom, Slack and Jamboard are examples of how members of research teams can engage in research as partners. Please let us know how these tools and activities are working for you so far.

---

First name ________________________________

Last name ________________________________

Email ________________________________

---

ZOOM

What device(s) are you using to join the Zoom meetings?

---

Device Type:

Check all that apply.

- ☐ Mac/Apple device
- ☐ Windows device (uses MicroSoft applications, such as Word, Excel)
- ☐ Other type of device

If "Other type of device" please describe: ________________________________

What device(s) are you using to join the Zoom meetings?

Check all that apply.

- ☐ Desktop computer
- ☐ Laptop computer
- ☐ Tablet or iPad
- ☐ Phone/iPhone/Android

## Zoom

**What is working well in participating in Zoom meetings so far?**

| | Yes | No |
|---|---|---|
| I am able to find and use the Zoom link. | ○ | ○ |
| I am able to use the chat feature in Zoom. | ○ | ○ |
| I am able to see everyone during the meeting. | ○ | ○ |
| I am able to hear everyone during the meeting. | ○ | ○ |

| | | |
|---|---|---|
| I am able to join the discussion. | ◯ | ◯ |
| I am getting to know the members of my group. | ◯ | ◯ |

Other things that are working well using Zoom: __________________________________

Are you having any problems participating in Zoom meetings?
◯ Yes
◯ No

If yes, please explain: __________________________________

**Slack**

**What is working well in participating on Slack so far?**

| | Yes | No |
|---|---|---|
| I can access Slack. | ◯ | ◯ |
| I can post a message. | ◯ | ◯ |
| I am getting to know who is who in my group. | ◯ | ◯ |
| I can set notifications to meet my preferences. | ◯ | ◯ |
| I can communicate with members of my group. | ◯ | ◯ |
| I can use the direct messaging feature. | ◯ | ◯ |
| I can reply to message threads and keep a discussion going. | ◯ | ◯ |
| I can reach the people I need to reach. | ◯ | ◯ |
| I can get my questions answered. | ◯ | ◯ |
| I can communicate my thoughts. | ◯ | ◯ |
| I feel confident posting messages. | ◯ | ◯ |
| I feel confident replying to messages | ◯ | ◯ |

Other things I appreciate about Slack: __________________________________

Are you having any problems participating on Slack?
◯ Yes
◯ No

If yes, please explain: __________________________________

## Jamboard

**What is working well in participating on Jamboard so far?**

| | Yes | No |
|---|---|---|
| I can access Jamboard. | ◯ | ◯ |
| I can manipulate the document tools. | ◯ | ◯ |
| I find Jamboard to be a meaningful way to share my ideas. | ◯ | ◯ |
| I find Jamboard a helpful way to see and understand others' perspectives. | ◯ | ◯ |
| I enjoy the discussion that Jamboard generates. | ◯ | ◯ |
| Jamboard helps me remember what we discussed. | ◯ | ◯ |

Other things I appreciate about Jamboard: ______________________________

Are you having any problems participating on Jamboard?
◯ Yes
◯ No

If yes, please explain: ______________________________

Please rate how your team is working for you:

I feel comfortable sharing information with my team during meetings and on Slack.
◯ Never
◯ Not often
◯ Sometimes
◯ Often
◯ Always

If you'd like, please explain your answer: ______________________________

People listen to my ideas and contributions respectfully during meetings and on Slack.
◯ Never
◯ Not often
◯ Sometimes
◯ Often
◯ Always

If you'd like, please explain your answer: ______________________________

I feel confident in making contributions to the conversation during meetings and on Slack.

- ◯ Never
- ◯ Not often
- ◯ Sometimes
- ◯ Often
- ◯ Always

If you'd like, please explain your answer:

__________________________________________

What would help you to stay fully engaged with your CIRCLE group at this point?

__________________________________________
