# Supplementary material for: Community case study of patient and clinician early engagement in research on multiple chronic conditions using an implementation guide
Source: Front Med (Lausanne). 2025 Oct 10;12:1642655. doi: 10.3389/fmed.2025.1642655 (PMC12549578; doi:10.3389/fmed.2025.1642655)
Supplement: Supplementary file 3 [file Data_Sheet_3.pdf]

Name of person sharing their lived experience:

**Type response here:**

Brief Bio: (age, gender,country of origin,race/ethnicity, occupation, education,employment status, health conditions you manage or help someone else manage, location in US, rural or city)

**Type response here:**

What Matters Most to you when (enter question here)
1.
2.
3.

Reflecting on a recent or significant health or healthcare experience, was anything pivotal about your experience that had a lasting impact on your ability to manage (name of topic)?

1.What circumstances prompted this experience?

**Type response here:**

2.What was said:

**Type response here;**

3.What was done:

Type response here:

4.How did this experience make you or others feel?

**Type response here:**

5.What impact did this experience have on you or others?

Type response here:

6.What could have been done differently for better outcomes?

**Type response here:**

7.What worked well for you or others?

**Type response here:**

8.What would you like other (roles of community members) you wish to learn and remember from your experience?

**Type response here:**

Group Learning Process:(capture the notes from what other members of the group learn or want others to remember from the experience that was shared today.

9. What do you believe others who hear or read your experience should learn from it:

**Type response here:**
