# Supplementary material for: Community case study of patient and clinician early engagement in research on multiple chronic conditions using an implementation guide
Source: Front Med (Lausanne). 2025 Oct 10;12:1642655. doi: 10.3389/fmed.2025.1642655 (PMC12549578; doi:10.3389/fmed.2025.1642655)
Supplement: Supplementary file 4 [file Data_Sheet_4.pdf]

CIRCLE APR Survey - Week 7

Thank you for being a member of our CIRCLE (cooperatively inspired research community for learning and engagement!) to work together with us on learning about research and the engagement of patients and clinicians in partnering for research.

We want to learn from your experience being on our project. We will be asking you to respond to a few questions during the last few weeks of the project. Please feel free to respond as honestly as you can. Your responses will help us understand more about how best to engage patients and clinicians in partnering for research.

Your responses will only be shared with our leadership team, Connie van Eeghen, Juvena Hitt, Jen Lavoie, and your facilitators.

What group/day meeting are you in?	◯ Monday<br>◯ Tuesday<br>◯ Wednesday<br>◯ Thursday<br>◯ Friday
Thinking back on this project over the past 7 weeks:	
What do you think is working well?	________________________
What questions do you have about our CIRCLE project?	________________________
What concerns do you have?	________________________
What suggestions do you have?	________________________
May we have your permission to follow up with you?<br><br>No matter which answer: We respect your time and energy.<br>Thank you for filling out this survey and helping us learn.	◯ Yes<br>◯ No
Name	____________________
Email	____________________
