# Supplementary material for: Community case study of patient and clinician early engagement in research on multiple chronic conditions using an implementation guide
Source: Front Med (Lausanne). 2025 Oct 10;12:1642655. doi: 10.3389/fmed.2025.1642655 (PMC12549578; doi:10.3389/fmed.2025.1642655)
Supplement: Supplementary file 5 [file Data_Sheet_5.pdf]

Appendix 5: Responses to "What suggestions do you have?"

| Suggestions to… | # (%) | Summary of respondent comments |
|---|---|---|
| None/All good | 18 (43) | |
| Improve engagement tools or processes | 14 (33) | ● Use the reflection with photo more systematically, include group dialogue from last session, provide a preview the week before (3 comments)<br>● Provide novice facilitators with more support (2)<br>● Make project goals more clear and specific (2)<br>● Plan for when no one volunteers<br>● More time to get to know other group members<br>● Better time management<br>● Balance challenging Shared Lived Experiences with positive experiences<br>● Provide an outline to support sharing the Lived Experience<br>● Provide a mailing option for surveys<br>● Provide a way to stay in touch after project end |
| Improve healthcare | 6 (14) | ● Take fatigue seriously<br>● Holistic care, cannabis products, virtual visits<br>● Address complaints systematically and support patient advocacy<br>● Include insurance companies in improvement<br>● Listen and reinforce our concerns<br>● Help patients know what to expect during visits |
| Improve technology | 4 (10) | ● Ensure that all have high quality broadband<br>● Provide more tech support before meeting start<br>● Reduce switching between interactive tools during meetings<br>● Provide more training on virtual platforms |
